# Fetal Growth Following Electronic Cigarette Use in Pregnancy

**DOI:** 10.3390/ijerph21091179

**Published:** 2024-09-04

**Authors:** Beth A. Bailey, Michelle Azar, Katherine Nadolski, Phoebe Dodge

**Affiliations:** 1 Department of Foundational Sciences, Central Michigan University College of Medicine, Mount Pleasant, MI 48859, USA; 2 Department of Family Medicine, Medical University of South Carolina, Charleston, SC 29425, USA; 3 Department of Pediatrics, Case Western Reserve University, Cleveland, OH 44106, USA

**Keywords:** electronic cigarettes, vaping, pregnancy smoking, in utero growth, birth size

## Abstract

Electronic cigarette (e-cig) use in pregnancy is common, but potential effects on fetal development are largely unknown. This study’s goal was to examine the association between e-cig exposure and fetal growth. Data were extracted from medical charts in this single-site retrospective study. The sample, excluding those with known tobacco, alcohol, illicit drug, opioid, and benzodiazepine use, contained women who used e-cigs throughout pregnancy and non-e-cig user controls. Fetal size measurements from second- and third-trimester ultrasounds and at birth were expressed as percentiles for gestational age. Following adjustment for confounding factors, in the second trimester, only femur length was significant, with an adjusted deficit of 11.5 percentile points for e-cig exposure compared to controls. By the third trimester, the femur length difference was 28.5 points, with the fetal weight difference also significant (17.2 points). At birth, all three size parameter differences between groups were significant. Significant size deficits were predicted by prenatal e-cig exposure, becoming larger and impacting more parameters with increasing gestation. While additional studies are warranted to confirm and expand upon these findings, this study adds to emerging data pointing to specific harms following e-cig exposure in pregnancy and suggests that e-cigs may not be a “safer” alternative to combustible cigarette smoking in pregnancy.

## 1. Introduction

Combustible cigarette (CC) use in pregnancy is the current leading cause of poor pregnancy outcomes [1], and while rates have been decreasing over the past two decades, 5.4% of U.S. pregnancies involve smoking tobacco [2]. Published human studies demonstrate immediate harms due to CC use in pregnancy, including fetal growth restriction, low birth weight (LBW), small for gestational age (SGA), preterm birth (PTB), pulmonary hypoplasia, increased rates of cesarean delivery, birth defects, admission to the neonatal intensive care unit, and fetal or neonatal death [3,4,5,6]. The use of electronic nicotine delivery systems, including electronic cigarettes (e-cigs), in pregnancy and their associated fetal and neonatal outcomes are less understood, but growing evidence suggests they are not insignificant and warrant further investigation. The American College of Obstetricians and Gynecologists (ACOG) recommends cessation of smoking in all forms during pregnancy [7], though studies have indicated the general public believes e-cigs to be safer alternatives in pregnancy [8]. While e-cigs eliminate the inhaling of smoke, which can cause specific damage, including decreased oxygen available to the fetus, many formulations still contain nicotine, the primary chemical cause of the fetal effects of maternal smoking, along with many other chemicals of unknown impact from the flavorings used.

The recent increased use and popularity of e-cigs has furthered the discussion of the specific harms of cigarettes in pregnancy, as up to 15% of pregnancies in the U.S. involve e-cig use [9]. Animal studies using zebrafish models have shown associations between exposure to e-cig aerosols and impaired bone and cartilage development [10]. Further, cinnamaldehyde flavoring in nicotine-free EC was associated with decreased cleithrum and bone length in zebrafish embryos [10]. In relation to prenatal exposure to e-cig vapor in mouse models, some studies demonstrated a significant reduction in birth weight [11,12], as well as decreased adult weight [13,14]. Human studies have found e-cig use in pregnancy to be linked to increased risk for babies being born SGA [15,16,17], LBW [16,18,19,20], and preterm [16,18,19]. 

There are presently no published human studies that examine the specific growth parameters affected by the use of e-cigs. However, fetal growth deficits following CC use in pregnancy have been examined across several studies. The literature supports that effects are largely influenced by the trimester of pregnancy corresponding with CC use. There are inconsistent findings across studies about whether CC use in the second trimester of pregnancy predicts decreased head circumference (HC), biparietal diameter (BPD), abdominal circumference (AC), mean abdominal diameter (MAD), or estimated fetal weight (EFW) [3,21,22,23,24,25,26,27,28,29,30,31]. Femur length (FL), however, was consistently found to be significantly reduced in the second trimester following maternal CC use [3,27,29]. When assessing fetal growth parameters in the third trimester, many of these same studies found decreased AC, MAD, and EFW in association with maternal CC use [21,22,23,26,27,30,31,32,33,34,35]. When examining FL in the third trimester, studies found either decreased FL [22,24,26,27,30,31] or no significant impact on FL [21,23,35,36] relative to maternal CC use in pregnancy. There has been no consistently observed relationship between HC and BPD in the third trimester with maternal CC use in pregnancy [21,22,23,24,26,27,30,36].

E-cig use is becoming more ubiquitous, especially among pregnant patients who may switch to e-cigs from CC use, perceiving it to be safer. However, the potential risks are largely unknown and may be similar to those associated with CC use, given that most e-cigs used by pregnant women contain nicotine and many other toxicants that have been implicated in negative fetal effects secondary to CC exposure [37]. Thus, it is necessary to understand how the use of e-cigs in pregnancy may impact fetal growth. Our pilot study aimed to examine the association between electronic cigarette exposure and fetal growth. Specifically, we investigated the following: Are effects evident at birth? If so, how early in gestation is growth impacted, and are the effects global, or do they impact only specific growth parameters?

## 2. Materials and Methods

### 2.1. Participants and Procedure

Participants in this retrospective cohort study were women who were receiving prenatal care at an academic obstetric practice that served patients from both the immediate semi-urban area and the larger surrounding rural region. Initial study eligibility included entering prenatal care during the study period (2018–2022), having a singleton pregnancy, being at least 18 years of age, entering prenatal care by 20 weeks gestation, and having completed the usual routine anatomy ultrasound between 18 and 22 weeks of pregnancy (per clinical guidelines), and an additional ultrasound with anatomical measurements taken between 30 and 34 weeks. A list of 1428 potentially eligible women was produced from an automated search of electronic medical records. Records for these women were then manually reviewed. For the parent study, women with various types of substance use were classified into exposure groups, in addition to non-substance-using women who made up the control group. Automatically eliminated were women who had not had any biological testing for substance use (n = 368). Women eligible for our study with biological assessment of substance use were compared with those for whom this was not performed, indicating no significant differences in any of the variables in Table 1. However, among the women in the larger parent study, those who completed biological testing were 18% more likely to have used opiates or cannabis during pregnancy compared to those not so assessed.

Participants were classified into the E-Cig group if they self-reported electronic cigarette use at any point during pregnancy or at delivery. Laboratory testing was ordered as clinically indicated, with at least one lab test available for all study participants. For most of the sample, this included at least one urine drug screen at entry to prenatal care (96%) and another urine drug screen at delivery (94%). Most had additional urine drug screens performed during pregnancy (90%) or had newborn cord blood testing results available (66%). All participants in the current analysis had no self-reported or biochemically confirmed alcohol, tobacco, or any other illicit drug use during pregnancy, with all laboratory testing negative for all tested substances. Additionally, the study participants did not have any prescriptions for opioids, benzodiazepines, or barbiturates during pregnancy. The control group met all these criteria, in addition to having no positive indicator for electronic cigarette use. A total of 20 women were classified into the E-Cig group, making them eligible for the current study, and all indicated use from the beginning of pregnancy through to at least the second-trimester ultrasound. A total of 171 women met the criteria and were classified into the control group.

A detailed manual review of electronic medical records was undertaken after the group assignment. Study data were abstracted to data collection sheets by medical student investigators, with regular team discussions and oversight by the senior investigator and a project manager to ensure data collection fidelity. Study data were entered into an electronic spreadsheet by the study manager, assisted by a trained undergraduate student and the medical student investigators. Frequent checks for entry accuracy were performed through double entry of ten percent of cases. All study procedures were approved by the Central Michigan University Institutional Review Board (study#: C-20-12 FETAL GROWTH) and included a waiver of informed consent. 

### 2.2. Variables

The primary predictor was electronic cigarette exposure status, grouped as E-Cig or control, as described. No information was consistently available about the amount or timing of electronic cigarette use, but all women in the E-Cig group reported regular use of electronic cigarettes at the time of the second-trimester ultrasound examination.

Study outcomes were derived from the reported results of the anatomy ultrasounds performed between 18 and 24 weeks gestation (second trimester) and between 30 and 34 weeks (third trimester). Outcome variables included estimated fetal weight, femur length, and head circumference, as well as the corresponding size measurements at birth (weight, length, head circumference). All measurements were converted to percentiles for gestational age.

Finally, additional information was collected to describe the study sample and to allow control of confounding. This included maternal background factors: age, race/ethnicity (coded as White non-Hispanic, African American, Hispanic, and Other), marital status (coded as married vs. single), highest level of education (collected as number of years but coded as high school graduate or less vs. any level of post-secondary education), and medical insurance (coded as Medicaid/none vs. private). Additional factors relative to the mother’s medical status that were used included parity, pre-pregnancy BMI (calculated and analyzed as a continuous variable), pregnancy weight gain, gestational age at first prenatal medical visit, development of gestational diabetes, an indication of hypertension (chronic or pregnancy-induced), gestational age at anatomy ultrasound, and infant sex.

### 2.3. Data Analysis

The study sample was intended to include all eligible electronic cigarette users; therefore, an a priori power analysis was not conducted. However, following data collection, a power analysis was performed to ensure that adequate statistical power was available to answer the primary research question. Based on the group sample sizes and using established means/standard deviations for U.S. populations, examination of estimated fetal weight revealed 80% power (at *p* < 0.05) to detect a 10% difference in weight at 20 weeks between the control and E-Cig groups.

Assumptions for all the statistical tests performed were met, including equality of variances between the two unequal-sized study groups. Chi-square analyses and *t*-tests examined the relationships between group membership and factors. Linear regression analysis was utilized to examine differences in the five fetal growth parameters between the control and E-Cig groups. Background factors significantly associated with group membership (*p* < 0.10) were entered stepwise, followed by the entry of the dichotomous electronic cigarette use group membership variable in the final step. Unstandardized regression coefficients from the final step represented the mean difference between the E-cig group and controls, with *p* < 0.05 considered significant. Data were managed and analyses were performed with IBM SPSS version 27.

## 3. Results

### 3.1. Background Differences between Study Groups

A total of 191 participants made up the final study sample: 20 women who used electronic cigarettes during pregnancy and 171 who did not. An analysis of background differences between the two study groups (Table 1) revealed that compared to the non-using control group, the E-Cig group was significantly more likely to be white, non-Hispanic, and single. Differences that approached significance (*p* < 0.10) were that e-cig users were more likely to have a high school education or less and to be using Medicaid or no medical insurance. All four of these factors were adjusted for in controlled analyses of study outcomes. 

### 3.2. Fetal and Newborn Size Differences between Study Groups

Size differences between the E-Cig and control groups are shown in Table 2. In the second trimester, after controlling for significant (*p* < 0.10) background differences, the E-Cig group had a significantly shorter average femur length than the controls, with a more than 11 percentile difference. However, there were no significant differences in estimated weight or head circumference. By the third trimester, the E-cig group was significantly smaller than the control group on all three size measurements, with femur length now decreased by over 28 percentile points. Differences remained significant at birth, with the E-Cig group significantly lighter, shorter, and with a significantly smaller head circumference compared to controls.

## 4. Discussion

The current study found that the use of electronic cigarettes during pregnancy predicted growth deficits evident as early as the second trimester of gestation, with length impacted early, and both weight and head circumference ultimately decreased following exposure. These findings are consistent with the findings from previous animal and human studies but also add additional insights. Several other studies report an association between prenatal e-cig exposure and birth weight in humans [16,17,18,19,20]. We have additionally demonstrated effects on size overall, with significant relationships between exposure and both newborn length and head circumference, suggesting this exposure may not just decrease body mass but could also impact bone growth. This is similar to what we have known for decades about the effects of utero combustible cigarette exposure, which have consistently been found to predict most anthropomorphic measurements, including length and head circumference at birth [38,39]. 

Our study also demonstrated that exposure to electronic cigarettes early in gestation may proximally impact fetal size, especially bone growth, as we found a relationship between exposure and femur length in the second trimester. By the third trimester, significantly decreased head circumference and overall weight were also identified among those exposed. Again, this is similar to what has been found following exposure to combustible cigarettes [3,22,24,27,29,30,31]. Based on our findings and preliminary work in other studies, electronic cigarettes do not appear to be a “safer” alternative to combustible cigarette use in pregnancy when considering potential impacts on fetal growth.

The current study has several strengths. Due to the use of existing data and the IRB-granted waiver of informed consent, our data were population-based, representing all women in prenatal care, with minimal potential for volunteer bias, a common concern with prospective research on substance use during pregnancy. In addition, we eliminated all women who smoked combustible cigarettes or used any other substances in pregnancy, allowing us to examine the potential effects of e-cigs on fetal growth independent of the known effects of other exposures. Finally, this study is the first that we know of to look at electronic cigarette use early in pregnancy in relation to specific fetal growth indices as early as the second trimester. 

Despite its strengths, this study is not without limitations. While the overall population from which this sample was drawn was fairly large, the number of electronic cigarette users who did not also smoke combustible cigarettes or use any other substances was limited. While we did have 80% power to detect moderate effect sizes, our conclusions would have been strengthened by a larger number of participants in our E-cig group. Related to this issue is the representativeness of the sample. Participants who used other substances during pregnancy were excluded to look more cleanly at the impact of electronic cigarettes in this smaller sample. This meant that our participants were not completely representative of all electronic cigarette users, as a sizable percentage do use other substances. Thus, we were unable to draw any conclusions about how the effects of electronic cigarettes on fetal growth may be enhanced or even mitigated by concurrent exposure to other substances. The generalizability of findings may also be limited due to the regional, relatively homogeneous single-site sample. A further limitation of the study was the methodology, with data coming retrospectively from medical charts. Substance use is often under-reported in the medical record during pregnancy due to many factors, including women not being asked, women unwilling to disclose use, and biological testing being intermittent at best. Thus, it is possible that some women who used electronic cigarettes were included in the control group and that women who used other substances were included in both groups, potentially underestimating the outcomes following electronic cigarette exposure in utero. Additionally, the amount, timing, and type of in utero electronic cigarette exposure may play a role in predicting growth outcomes; however, this information was unavailable in the current study. Finally, this study was observational in nature and, as such, does not permit cause-and-effect conclusions. Further research is needed to address many of these limitations and add validity to the current results.

The clinical and public health relevance of our findings are important to consider. Many women, and even medical professionals, believe that e-cigs may be a better alternative to combustible cigarettes during pregnancy. Based on our findings and those of other recent studies, that does not appear to be the case. Even use early in pregnancy, as we demonstrated, may cause harm. Pregnant women should be encouraged to eliminate all forms of smoking during pregnancy, and clinical advice and public health messaging should be consistent and clear in this recommendation to promote the healthiest pregnancies possible. 

## 5. Conclusions

In this preliminary study, significant fetal size deficits were associated with prenatal electronic cigarette exposure and were evident as early as the second trimester. Differences in fetal size between pregnancies involving electronic cigarette exposure and those that did not were larger and involved more growth parameters with increasing gestation, and by birth involved all three parameters studied: weight, length, and head circumference. Given the small sample size, additional study is needed to confirm these findings and to explore the potential roles of timing and amount of exposure, as well as specific flavorings and ingredients in e-cigs used. However, this study adds to emerging data pointing to specific harms following electronic cigarette exposure in pregnancy and suggests that e-cigarette use may not be a “safer” alternative to combustible cigarette smoking in pregnancy.

## Figures and Tables

**Table 1:** Background differences between electronic cigarette users and non-users.

	Controls (n = 171)	E-Cig Users (n = 20)	*p*
Maternal age at delivery (yrs)	25.4 (4.8)	24.4 (4.2)	0.409
Maternal race (% white, non-Hispanic)	41.5%	78.9%	0.002
Maternal marital status (% single)	67.1%	100%	0.003
Maternal education (% HS grad or less)	58.5%	78.9%	0.083
Maternal insurance (% Medicaid/none)	68.4%	89.5%	0.056
Parity	1.1 (1.2)	1.0 (1.1)	0.544
Pre-pregnancy BMI	28.4 (7.1)	28.2 (7.5)	0.909
Pregnancy weight gain (lb)	23.1 (12.0)	29.6 (16.2)	0.226
Gestational age 1st prenatal visit (wk)	14.6 (4.0)	16.4 (5.0)	0.184
Diabetes (% existing/gestational)	11/7%	0.0%	0.115
Hypertension (% chronic/pregnancy induced)	17.0%	5.3%	0.185
Gestational age 2nd trimester ultrasound (wk)	20.3 (1.7)	20.3 (1.4)	0.919
Gestational age 3rd trimester ultrasound (wk)	34.2 (3.5)	34.0 (2.8)	0.836
Gestational age at delivery (wk)	38.6 (2.2)	38.1 (3.9)	0.569
Fetal gender (% male)	53.8%	63.2%	0.437

**Table 2:** Adjusted ^1^ mean percentile size differences between those with electronic cigarette exposure compared to those without electronic cigarette exposure.

	Unstandardized Regression Coefficient (SE)	*p* ^2^
Second-Trimester Ultrasound (18–22 wks)		
Estimated fetal weight	−0.01 (6.5)	0.499
Femur length	−11.5 (6.6)	0.042
Head circumference	−4.5 (6.3)	0.240
Third-Trimester Ultrasound (30–24 wks)		
Estimated fetal weight	−17.2 (8.9)	0.028
Femur length	−28.5 (9.3)	0.001
Head circumference	−11.1(8.6)	0.048
Birth		
Weight	−12.8 (6.5)	0.025
Length	−19.3 (6.9)	0.003
Head circumference	−13.3 (7.4)	0.037

^1^ Regression coefficient controlled for maternal race, marital status, education, and insurance. ^2^ One-tailed test.

## Data Availability

Restrictions apply to the availability of these data. Data were obtained from HIPAA protected medical records of the Covenant Health System and are only available directly from that organization.
